# Low birthweight in rural Cameroon: an analysis of a cut-off value

**DOI:** 10.1186/s12884-018-1663-y

**Published:** 2018-01-15

**Authors:** Valirie Ndip Agbor, Chobufo Ditah, Joel Noutakdie Tochie, Tsi Njim

**Affiliations:** 1 Ibal Sub-divisional Hospital, Oku, Northwest Region, Bamenda, Cameroon; 2 Doctors Without Borders-French Section (MSF-F), Calais, France; 3 Department of Surgery and sub-Specialties, Faculty of Medicine and Biomedical Sciences, University of Yaoundé I, Yaoundé, Cameroon; 4 Health and Human Development Research Group (2HD), Douala, Cameroon; 5 Centre for Global Health and Tropical Medicine, Nuffield Department of Medicine, University of Oxford, Oxfordshire, UK

**Keywords:** Cut-off value, Low birthweight, Adverse foetal outcomes, Rural Cameroon

## Abstract

**Background:**

Low birthweight (LBW) is a major predictor of early neonatal mortality which disproportionately affects low-income countries. WHO recommends regional definitions for LBW to prevent misclassifications and ensure appropriate care of babies with LBW. We conducted this study to define a clinical cut-off for LBW, and to determine the predictors and adverse foetal outcomes of LBW babies in a rural sub-division in Cameroon.

**Methods:**

We conducted a retrospective register analysis of 1787 singleton deliveries in two health facilities in the Northwest Region of Cameroon. Records with no birthweight or birthweight less than 1000 g, babies born before arrival, multiple deliveries and deliveries before 28 weeks gestation were excluded from this study. The 10th percentile of birthweights was computed to obtain a statistical cut-off value for the LBW. To assess the clinical significance of the newly defined cut-off value, we compared the prevalence of adverse foetal outcomes between LBW (birthweight <10th percentile) and heavier babies (birthweight ≥10th percentile) in our study population.

**Results:**

The 10th percentile of the birthweights was 2700 g. Preterm delivery was the lone predictor of LBW (aOR = 2.0, 95% CI = 1.3–3.1; *p =* 0.001). LBW babies were more likely to be stillborn (OR = 9.6; 95% CI = 4.2–21.6; *p* < 0.001) or asphyxiated at the 5th minute (OR = 2.0; 95% CI = 1.2–3.3; *p* = 0.006), compared with heavier babies. Also, 6.1% of babies who had a birthweight between 2500 and 2700 g were more likely to be stillborn compared to heavier babies.

**Conclusion:**

This study suggests that the clinical cut-off for LBW in this rural community is 2700 g; with 6.1% of babies born with LBW probably receiving inadequate care as the traditional cut-off value of 2500 g proposed by WHO is still used to define LBW in our setting. Further studies are necessary to define a national cut-off value for harmonisation of LBW definitions in the country to prevent misclassifications and ensure appropriate neonatal care.

## Background

The World Health Organisation (WHO) defines low birthweight (LBW) as the weight of a neonate below 2500 g at birth, often corresponding to the 10th percentile for its gestational age [1]. Globally, 20 million neonates are born with LBW, 95% of whom reside in developing countries [1]. Africa, carries the second highest burden of LBW (14.3%) after Asia (18.3%) [1]. LBW babies are about 20-fold more likely to develop complications compared with heavier babies. Also, LBW is a major predictor of mortality among infants in low-income countries, especially during the neonatal period [2]. LBW-associated morbidity is further highlighted as it is a significant determinant of failure to thrive, poor cognitive development and chronic diseases in both childhood and adulthood [3, 4]. Globally, LBW is a good indicator of public health problems like chronic maternal illness, malnutrition, hard-work and poor antenatal care [1]. It has become increasingly evident that the traditional cut-off value of 2500 g advocated by WHO to define LBW may not be generalizable to every settings. For instance, some countries like Sri Lanka with a high incidence of newborns weighing less than 2500 g, do not have an associated high mortality rate [5]. To this effect, WHO has endorsed the definition of a local cut-off value for LBW for every country [1]. However, local data to define LBW in Cameroon, and Africa at large remains meagre. This is worrisome as some babies might not receive appropriate care due to misclassification [6]. Njim and colleagues have conducted the lone study so far, in a sub-urban area, to define a practical cut-off for LBW in Cameroon using the 10th percentile [6]. With the aim of contributing to the available body of evidence on LBW in Cameroon, we conducted this study to: define a clinical cut-off for LBW making comparisons with that provided by Njim et al.; and to identify the predictors and adverse outcomes of LBW babies in a rural setting in Cameroon.

## Methods

### Study design, duration and settings

The study methodology has been described in a previous study [7]. Briefly, this was a retrospective register analysis of deliveries conducted between January 1st, 2009 and December 31st, 2016; an 8-year period. This study was conducted in two health facilities: Oku District Hospital and Kevu integrated health centre, in the Oku Sub-division, Northwest Region, Cameroon. These facilities conduct the greater majority of deliveries in this health district. This Sub-division consist of 93,000 inhabitants with farming being the main activity of the people.

### Participants and data collection

All delivery records during the study period were reviewed. Eligible for inclusion were all singleton hospital deliveries. We excluded records with no birthweight or birthweight less than 1000 g, babies born before arrival, multiple deliveries and deliveries before 28 weeks gestation. Out of the 2343 delivery records reviewed, 1787 were retained, giving a response rate of 76.4%.

We collected data on sociodemographic and clinical characteristics of all included mother-neonate pairs which included: age, marital status, gravidity, parity, gestational age, human immunodeficiency virus (HIV) serologic status and gender of the neonate. Foetal outcome parameters such as: birthweight, fifth minute Apgar score, and stillbirth were also collected. Variables were categorised as shown in Table 1. For a comprehensive analysis, parity was categorised into: primiparity – Yes (one previous term delivery) or No (more than one previous term delivery).

**Table 1:** Definition of operational variables

Gravidity	1. Primigravida (Women at their first pregnancy)
	2. Multigravida (2–4 pregnancies)
	3. Grand multigravida (> 4 pregnancies)
Parity	1. Primiparous (Women who had one previous term delivery)
	2. Multiparous (2–4 previous term deliveries)
	3. Grand multiparous (> 4 previous term deliveries)
Gestational age	1. Preterm delivery: Delivery from 28 to 36 weeks of gestation
	2. Term delivery: Delivery from 37 to 42 weeks of gestation
	3. Post-term delivery: Delivery above 42 weeks of gestation
Apgar score at fifth minute	Neonatal asphyxia. Yes (< 7) versus No (≥ 7)

### Statistical analysis

Data were entered and analysed using the Statistical Package for Social Sciences (SPSS) version 20.0. Categorical variables are presented as frequencies, proportions and percentages, while continuous variables are presented as means (Standard Deviation) and median (Inter-Quartile Range), where appropriate. The 10th percentile of the birthweights was computed to obtain the cut-off for LBW in our study population. The kappa Cohen coefficient was used to evaluate levels of agreement between the newly defined cut-off, the traditional cut-off value and the local cut-off proposed by Njim and colleagues [6]. To assess the clinical significance of the newly defined cut-off value, we evaluated the likelihood of LBW babies (babies with birthweights below the newly defined cut-off) to develop adverse foetal outcomes compared with heavier babies. Associations between categorical variables were investigated using the Chi square or Fisher’s exact tests where appropriate. The degree of association between the categorical variables was estimated using the odd’s ratio (OR) and corresponding 95% confidence intervals (CI). To determine independent predictors of LBW, a multivariate logistic regression model was built using variables with *p*-values < 0.25. Two sided p-values less than 0.05 were considered statistically significant.

### Ethical considerations

Ethical approval for the conduct of this study was granted by the Regional delegation of public Health of the Northwest Region of Cameroon and by the directorate of both partaking hospitals.

## Results

Overall, 1787 records were eligible for this study. Maternal ages ranged from 14 to 49 years, with a mean age of 26 ± 6.5 years. Over three quarters of women in our study were married by the time of delivery. Also, one quarter of the women were delivering for their first time. There was an equal number of male as female neonates (50.6% versus 49.4%; Table 2). The 10th percentile for birthweight was equivalent to 2700 g. There was a moderate level of agreement (kappa = 0.54) with the traditional cut-off value of 2500 g proposed by WHO [1]. However, our cut-off strongly agreed (kappa = 0.997) with the cut-off for LBW reported by Njim et al. in a sub-urban Region of Cameroon [6]. After controlling for maternal HIV status, primiparity, maternal age and gender of the infant (Table 3), preterm delivery was the lone predictor of LBW in multivariate analysis [adjusted odd’s ratio (aOR) = 1.5; 95% CI = 1.1–2.2; *p =* 0.001; Table 4]. Also, compared with heavier babies, LBW babies were more likely to be stillborn (OR = 9.6; 95% CI = 4.2–21.6; *p* < 0.001) or suffer from birth asphyxia at the fifth minute of life (OR = 2.0; 95% CI = 1.2–3.3; *p =* 0.006), Table 5. About 6.1% (163/1787) of babies with birthweights less than 2700 g were found within the grey zone (2500 to < 2700 g). These babies were more likely to be stillborn compared with heavier babies (OR = 6.4; 95% CI = 2.2–18.5; *p =* 0.006; Table 6).

**Table 2:** Characteristics of the study population

Variable	Low birthweight		Total N (%) = 1787
	Yes, *n* = 180 (%)	No, *n* = 1607 (%)	
Maternal age			
Mean (SD)	25.9 (7.5)	26.0 (6.5)	26.0 (6.5)
Median (IQR)	25.0 (19.0–32.0)	25.5 (21.0–30.5)	25.0 (21.0–30.5)
Marital status			
Single	60 (33.5)	374 (23.4)	434 (24.4)
Married	119 (66.5)	1226 (76.6)	1346 (75.6)
Gravidity			
Primigravida	65 (36.3)	363 (23.2)	428 (24.5)
Multigravida	62 (34.6)	782 (50.0)	844 (48.3)
Grand multigravida	52 (29.1)	421 (26.9)	474 (27.2)
Parity			
Primiparous	66 (36.9)	375 (24.0)	441 (25.3)
Multiparous	63 (35.2)	774 (49.4)	837 (47.9)
Grand multiparous	50 (27.9)	417 (26.6)	468 (26.8)
Gestational age			
Term	89 (54.9)	933 (63.4)	1022 (62.6)
Preterm	69 (42.6)	462 (31.4)	532 (32.6)
Post term	4 (2.5)	76 (5.2)	80 (4.9)
Maternal HIV status			
Positive	11 (6.1)	70 (4.4)	81 (4.5)
Negative	169 (93.9)	1535 (95.6)	1705 (95.5)
Gender of infant			
Male	92 (52.0)	810 (50.5)	902 (50.6)
Female	85 (48.0)	795 (49.5)	881 (49.4)

*HIV* Human immunodeficiency virus, *N* frequency, *SD* standard deviation, *IQR* interquartile range

**Table 3:** Predictors of low birth-weight on univariate analysis

Variable	LBW, n (%)	Total, N	OR (95% CI)	*p-*value
Maternal age				
< 20 years	48 (14.0)	365	1.6 (1.1–2.3)	0.008
≥ 20 years	129 (9.1)	1422		
Marital status				
Single	58 (13.5)	430	1.6 (1.0–2.7)	0.034^*^
Married	113 (8.4)	1343		
Primiparity				
Yes	66 (15.0)	441	1.9 (1.3–2.7)	< 0.001^*^
No	113 (8.7)	1304		
Gestational age				
Term	88 (8.7)	1022		< 0.001^*^
Preterm	69 (13.0)	531	2.2 (1.5–3.2)	
Post term	4 (5.0)	80	0.3 (0.1–1.4)	
Maternal HIV status				
Positive	11 (13.6)	81	1.4 (0.7–2.7)	0.274
Negative	169 (7.0)	1704		
Gender of infant				
Female	85 (9.7)	880	1.0 (0.7–1.4)	0.225
Male	92 (10.2)	902		

*OR* Odd’s ratio, *CI* Confidence interval, *n* Frequency, *LBW* low birthweight, * significant variables

**Table 4:** Multivariate analysis of predictors of low birth-weight

Variable	LBW, n (%)	Total, N	aOR (95% CI)	*p-*value
Maternal age				
< 20 years	48 (14.0)	365	1.0 (1.0–1.1)	0.51
≥ 20 years	129 (9.1)	1422		
Marital status				
Single	58 (13.5)	430	0.8 (0.5–1.1)	0.180
Married	113 (8.4)	1343	–	
Primiparity				
Yes	66 (15.0)	441	1.1 (0.1–8.4)	0.960
No	113 (8.7)	1304	–	
Gestational age				
Term	88 (8.7)	1022	–	
Preterm	69 (13.0)	531	1.5 (1.1–2.2)	0.001^*^
Post term	4 (5.0)	80	0.6 (0.2–1.6)	0.277
Sex of infant				
Female	85 (9.7)	880	1.1 (0.8–1.5)	0.628
Male	92 (10.2)	902	–	

*aOR* adjusted odd’s ratio, *CI* Confidence interval, *n* Frequency, *LBW* low birthweight, * significant variables

**Table 5:** Adverse outcomes of neonates with low birthweight

Variable	LBW, n (%)	No LBW, n (%)	OR (95% CI)	*p-*value
Neonatal asphyxia (5th min Apgar)				
Yes, n (%)	10 (6.0)	45 (2.8)	2.2 (1.1–4.4)	0.033^*^
No, n (%)	156 (94.0)	1543 (97.2)		
Still birth				
Yes, n (%)	12 (6.7)	12 (0.8)	9.5 (4.2–21.5)	< 0.001^*^
No, n (%)	168 (93.3)	1594 (99.3)		

*OR*, Odd’s ratio, *CI* Confidence interval, *n* Frequency, *LBW* low birthweight (< 2700 g), * significant variables

**Table 6:** Adverse outcome of neonates with birthweight of 2500 to < 2700 g

Variable	BW_1_, *N* = 163	BW_2_, *N* = 1553	OR (95% CI)	*p-*value
Neonatal asphyxia (5th min Apgar)				
Yes, n (%)	6 (11.8)	45 (88.2)	2.1 (0.9–5.2)	0.126
No, n (%)	98 (6.0)	1543 (94.0)		
Still birth				
Yes, n (%)	5 (29.4)	12 (70.6)	6.4 (2.2–18.5)	0.003*
No, n (%)	104 (6.1)	1594 (93.9)		

*BW*_*1*_ Birthweight within the category 2500 to < 2700 g, *BW*_*2*_ Birthweight ≥ 2700 g, *N* absolute frequency, *CI* confidence interval, * significant variables

## Discussion

The locally defined clinical cut-off for LBW in this community was found to be 2700 g, with preterm delivery being the lone predictor of LBW. Also, LBW babies were more likely to be stillborn and asphyxiated at birth. More so, babies within the grey zone were more likely to be stillborn compared with heavier babies.

To the best of our knowledge, only one study in sub-Saharan Africa has defined a local cut-off for LBW [6]. Local cut-off values for LBW have been defined in some developed countries, and ranges from 2750 g in the US in 1992 [8] to 3000 g in Denmark in 2007 [9]. Our cut-off for LBW strongly agreed with the 2600 g previous reported by Njim and collaborators in 2015, in sub-urban Cameroon [6]. These results which are similar suggest that the cut-off for LBW in Cameroon proposed by WHO might not be appropriate for use in clinical care, as vulnerable babies within the grey zone (weights ≥ 2500 g but < 2700 g) are probably not receiving adequate care as they are rather regarded to have a normal birthweight. In this study, 6.1% of the babies were found in this grey area. LBW is a significant predictor for neonatal mortality [10]. Such misclassifications are likely contributors to the overall neonatal mortality. Indeed, babies born with birthweights within this grey zone were about six times more likely to be stillborn compared with heavier babies. This emphasises the need for more studies within the country to harmonise LBW definitions for the institution of appropriate public health policies to address the problem.

Preterm delivery was the lone predictor of LBW in this study. This finding is consistent with other reports from sub-Saharan Africa [6, 11, 12]. It is obvious that preterm infants will have a LBW, as they are not privileged to attain the required term for a normal weight. Preterm deliveries have been associated with poor antenatal care and chronic maternal diseases like HIV [6, 13, 14]. Nevertheless, we found no significant association between being an adolescent, a single mother, a female neonate and LBW. Also, the first pregnancy and delivery, and a positive maternal HIV status did not predict LBW. This finding corroborated with that of Njim et al. [6]. In contrast to observations made by Abubakari et al. [15], we did not find a significant association between primiparity and a female neonate with LBW, probably explained by the fact that the authors did not account for confounders in their study. Finally, LBW was significantly associated with neonatal asphyxia at the 5th minute of life and stillbirth. This finding is consistent with other African series [6, 16]. This reiterates reports by Koum and colleagues which depict LBW as a major contributor to the early neonatal mortality burden in Cameroon [10], and therefore the need to implement evidence-based measures, like defining a local cut-off value for LBW, to provide appropriate care to these infants. Providing care to LBW babies using evidence-based methods might be key in achieving the third goal of sustainable development [17].

Despite its limitations, birthweight remains a relatively reliable parameter to determine the risk of neonatal mortality. This is especially true in rural communities where the use of first trimester ultrasonography for gestational dating is practically non-existent. However, the records of gestational ages (based on the clients’ last menstrual periods) entered into the delivery registers might not be accurate as it is subject to recall bias. The length of the gestation is also crucial in predicting neonatal morbidity and mortality [18], and simply adopting the new cut-off value of 2700 g for the LBW might have limited value in curbing neonatal morbidity and mortality in these rural communities.

### Study limitations

Being a retrospective study, we had no influence on the quality of data entered into the delivery registers. The risk of other neonatal complications like neonatal infection, admission to the neonatal unit, and hypoglycaemia, for example, could not be evaluated due to unavailability of relevant data. In addition, predictors of LBW such as use of recreational drugs, smoking and alcohol consumption during pregnancy could not be assessed due to lack of data collected on these variables in our delivery registers. This study was not designed to identify constitutional causes of LBW (genetic predisposition), highlighting the need for further research in this domain. However, with the large sample size (1787 newborns) and a robust statistical analysis controlling potential confounders, we ruled out several potential biases in the results obtained.

## Conclusion

We have demonstrated that the clinical cut-off for LBW in this rural community is 2700 g, with preterm delivery being the sole determinant of LBW. LBW babies were more likely to be stillborn or asphyxiated at the fifth minute of life. A significant proportion of these babies may be receiving inadequate neonatal care as the traditional cut-off of 2500 g proposed by the WHO is still used to define LBW in this rural area. However, more data is needed through a prospective study design with larger sample size to define a cut-off value for LBW over the national territory; as this will guide the implementation of effective preventive strategies to curb the morbidity and mortality associated with LBW in Cameroon. While pursuing the validation of a cut-off value for LBW on a larger scale in Cameroon, it is prudent to adopt our current cut-off value for LBW in this rural community.

## Abbreviations

aOR: Adjusted odd’s ratio
CI: Confidence interval
HIV: Human Immunodeficiency Virus
LBW: Low birth weight
OR: Odd’s ratio
US: United States
WHO: World Health Organisation
